# Building healthy beginnings: A qualitative descriptive study of midwives’ role in identifying and promoting mentally healthy mothers in Australia

**DOI:** 10.1177/22799036261434100

**Published:** 2026-03-26

**Authors:** Lesley Pascuzzi, Karen Heslop, Helen Skouteris, Zoe Bradfield

**Affiliations:** 1 School of Nursing, Faculty of Health Sciences, Curtin University, Perth, WA, Australia; 2 Health and Social Care Unit, School of Public Health and Preventive Medicine, Monash University, Melbourne, VIC, Australia; 3 Department of Nursing and Midwifery Education and Research, King Edward Memorial Hospital, Women’s and Newborn’s Health Service, Perth, WA, Australia

**Keywords:** mentally healthy mothers, maternal mental health promotion, qualitative descriptive study, midwives’ scope of practice, public health

## Abstract

**Background:**

Good mental health positively impacts public health by improving maternal and neonatal outcomes. Women benefit from routine care and health promotion provided by midwives. However, there is no documented evidence regarding midwives’ perspectives on their public health role in supporting mentally healthy mothers and little is known about midwives’ understanding and practices in this area. To address this evidence gap, the aim of this study was to qualitatively explore perspectives of identifying and promoting maternal mental health and wellbeing.

**Design and methods:**

A qualitative descriptive approach was used. Data were collected via online 1:1 interviews or a focus group. Data were analyzed using inductive thematic analysis.

**Results:**

Ten midwives participated. Six semi-structured interviews and one focus group of four midwives were conducted. Five themes were generated: The Well Woman; Scope of Practice; Impact of Current Maternity System; Continuity of Care and Perinatal Mental Health Literacy.

**Conclusions:**

Findings indicate Australian midwives are well-placed to promote maternal mental health within routine practice but have limited resources. Midwives described promoting mental health as being within their role and scope of practice, reporting barriers including societal views, priorities, and systemic pressures of time and limited access to both publicly funded and private continuity of care models. Our findings suggest midwives in Australia are well-placed but not equipped to promote maternal mental health at present.

## Introduction

In Australia, mental health conditions are the leading cause of disease burden to women of childbearing age and public health data estimate that one in five women are affected.^1^ Perinatal mental health refers to the mental health of women during pregnancy and the first year postpartum.^2^ National perinatal guidelines prioritize mental healthcare by screening for risk and identification of common mental health difficulties including anxiety and depression^3^ influencing the direct clinical practice of perinatal healthcare professionals including midwives.

**Figure 1.**
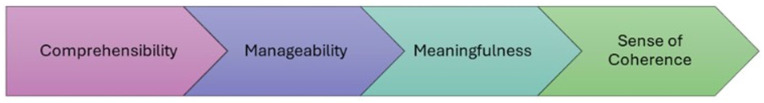
Sense of coherence.

The World Health Organization (WHO) defines maternal mental health as “a state of well-being in which a mother realizes her abilities, can cope with the normal stresses of life, can work productively and fruitfully, and contribute to her community.”^4^ The transition to motherhood is reported to be significant in the life span, a time when optimal mental health will positively impact maternal and neonatal outcomes.^3,5^ Conversely, child-bearing women experiencing perinatal mood and anxiety disorders (PMADs) may adversely affect the developing maternal-infant relationship and the infant’s early development.^6^ Therefore, prevention of PMADs and promotion of maternal mental health and emotional wellbeing is prioritized within public health guidelines.^3^ In addition to illness-oriented screening approaches, strengths-based frameworks are increasingly recognized within public health. Salutogenesis, introduced by Antonovsky,^7^ conceptualizes health along a continuum rather than as the absence of disease. Central to this framework is Sense of Coherence (SOC), comprising comprehensibility (making sense of experiences), manageability (perceived access to resources), and meaningfulness (motivation to engage) shown in Figure 1. Higher SOC has been associated with improved coping and psychological wellbeing, including in perinatal populations.^8,9^ A salutogenic orientation aligns with midwifery philosophy,^10^ which supports and normalizes physiology, relational care, and supporting women’s inherent capacity for health. Exploring midwives’ perspectives of promoting mental health within routine care can therefore be understood within this broader strengths-based public health framework.

Around the world, midwives are essential to the provision of maternity care to optimize maternal and neonatal outcomes.^11^ In Australia, midwives are the largest primary maternity workforce, providing expert care to most of the >300,000 women who give birth every year.^12^ The profession of midwifery is culturally positioned as a trusted, relational profession grounded in woman-centered care and continuity across the childbearing continuum.^10^ Midwives provide care to most women during pregnancy, birth, and the early postnatal period, often forming sustained relationships that extend beyond episodic clinical encounters. This relational model of care supports high levels of trust, disclosure, and engagement, positioning midwives as key public health practitioners with unique opportunities to influence maternal mental health and emotional wellbeing. Within this context, midwives’ scope of practice extends beyond clinical surveillance to include health promotion, education, and advocacy, making their perspectives particularly salient when exploring how mental health promotion is understood and enacted within routine maternity care.

High-quality maternity care plays a central role in strengthening public health, recognizing both the core health promotion skills of the midwifery workforce and the influential relationships established between a woman and her midwife.^13,14^ Herrman et al.^4^ describe this relational role succinctly, noting that “every interaction a midwife has with a service user is an opportunity to either promote or demote mental health.” Despite this, existing research examining midwives’ perspectives predominantly reflects the illness-oriented focus of perinatal guidelines,^3^ with emphasis on screening, referral, and management of perinatal mood and anxiety disorders.^15–19^ Far less is known about how midwives conceptualize and enact mental health promotion for women who are not experiencing diagnosable mental illness.

Professional organizations in Australia endorse midwives working to full scope to strengthen maternal mental health.^13,14^ However, reported barriers, including limited confidence, gaps in knowledge and competing organizational priorities constrain this in practice.^15^ The prevailing model of employment of midwives is fragmented models of maternity care where women are cared for by multiple clinicians across pregnancy and the postnatal period.^20^ A mere 28% of women have access to midwifery continuity of care models^21,22^ with variation in access outside metropolitan areas. Maternal mental health and emotional wellbeing is positively influenced by continuity of midwifery care models^23^ to reduce mental health burden and prevent the development of PMADs. Continuity of midwifery care is desired by women in Australia^24^ and there is evidence to support more favorable mental health outcomes when compared to fragmented midwifery care.^23^ Although midwives are the largest maternity workforce and are professionally positioned to provide holistic, relational care, fragmented models can limit opportunities for continuity, trust-building, and proactive psychosocial engagement.^23^ These structural constraints may contribute to professional barriers previously reported in the literature.

Within this context of high perinatal mental health need and a fragmented maternity care system, little is known about how mental health promotion is understood and enacted by midwives within these structural conditions, highlighting a critical public health evidence gap that this study seeks to address. The aim of this study therefore was to explore midwives’ perceptions of identifying and promoting mental health and emotional wellbeing when caring for women in the perinatal period. The study was guided by these questions:

1. How do midwives identify and describe emotional wellbeing in the perinatal period?
2. What experiences can midwives offer as examples of promoting mental health and emotional wellbeing within routine maternity care?
3. Do midwives have solutions to strengthen their ability to work within a mental health promotion model within the perinatal period?

## Design and methods

A qualitative descriptive approach was selected because the aim of this study was to obtain a clear, practice-focused description of midwives’ perspectives on identifying and promoting maternal mental health and emotional wellbeing within routine maternity care. Qualitative description is a methodology particularly well suited to midwifery research internationally^25,26^ and in Australia^27,28^ that seeks to understand *what* participants perceive and experience, without imposing high levels of theoretical or interpretive abstraction.^29^ A pragmatic framework^30^ guided this study, supporting the generation of practice-focused, real-world insights. Although the study adopted an inductive qualitative descriptive approach and was not designed to test a predefined theoretical model, it was informed by a broader salutogenic orientation. The concept of Sense of Coherence^7^ provided a conceptual framework for understanding how midwives describe supporting women’s emotional wellbeing, particularly in relation to comprehensibility (knowledge), manageability (resources), and meaningfulness (professional motivation). The reporting of this study conforms to the COREQ guidelines for reporting qualitative studies.^31^ A completed checklist is presented in Supplemental File 1.

This approach enabled midwives’ accounts to be reported in language that remains close to participants’ own descriptions, producing findings that are readily accessible and directly relevant to explore this under-researched area while minimizing interpretation. Consistent with the study’s aim to inform future strategies to strengthen maternal mental health promotion within maternity care, qualitative description supported the generation of findings to inform subsequent stages of the PhD project. This study received ethical approval from Curtin University Human Research Ethics Committee (HRE2024-0135) on 5th April 2024.

### Participant recruitment

Midwives were recruited to the study via relevant professional social media sites (Facebook, X, Instagram, and LinkedIn) between April and May 2024. Social media is an increasingly popular recruitment tool in qualitative research^32^ with various platforms offering instant access to potential participants. Respondents were able to express their interest to participate in a semi-structured interview or a focus group if they identified as a midwife or midwifery leader delivering maternity care to women in Australia within the last 10 years. Purposive sampling was used to recruit midwives with relevant professional experience in maternity care and mental health promotion. Recruitment continued until data sufficiency was achieved, defined as the point at which no new codes, patterns, or substantive insights relevant to the study aims were identified in successive interviews and the focus group. No participants refused to participate or dropped out. Adequacy of the sample was assessed through ongoing concurrent analysis, with repetition observed across participants’ descriptions of key concepts, experiences, and perceived barriers and enablers. Given the study’s qualitative descriptive design and its focus on generating practice-relevant insights rather than theory development, the final sample size was considered sufficient to address the research questions.

### Data collection

Six midwives participated in semi-structured interviews and four midwives participated in a 1-h focus group between May and June 2024. Interviews and focus groups were facilitated by the full-time PhD researcher (first author). Interview and focus group questions (Supplemental File 2) were guided by the research aims identified and were piloted for feasibility. Pilot responses were not included in data analysis. Participating midwives provided written informed consent prior to participation. All data collection activities were hosted on MS Teams and audio/video recorded where possible. Interview duration varied from 34 to 65 min. Focus group duration was 60 min. On completion, transcripts were checked for accuracy by the PhD researcher. Participants were largely at home during participation, with one midwife attending from her workplace setting. Five participants were unknown to the researcher at the time of interview, and one participant shared the same clinical workplace as the first author. Throughout all activities, the participant(s) and the researcher were the only attendees present during data collection. No repeat interviews were conducted. The PhD researcher (she/her) has experience conducting clinical interviews within mental health settings and facilitating antenatal parent education with families in pregnancy. Throughout the research process, maintaining a research journal^33^ and engaging with co-authors/supervision team ensured any personal biases affecting the quality of data collection and analysis were documented and discussed. The co-author/supervision team consisted of an experienced midwife researcher, mental health nurse researcher and a health promotion researcher experienced in developmental psychology and implementation science. Furthermore, including verbatim transcription with accuracy checking, reflexive journaling, multidisciplinary team supervision discussion during the analysis phase, and participant member checking were strategies employed to enhance data quality and ensure the trustworthiness of the data collected.

### Data analysis

Inductive thematic analysis was selected to analyze the transcript data. Thematic analysis^34^ is a six-phase framework to identify, analyze, and report patterns (themes) within data sets. To successfully work with data, comprehensive understanding of transcript content develops familiarity to generate initial codes. Grouping of codes that have supporting quotations within the data set allow themes to be generated that can be reviewed, revised and finalized.^35^ All transcripts were coded by the PhD researcher using NVivo software (version 14). To enhance rigor and trustworthiness, emerging codes and themes were discussed and reviewed during regular supervision meetings with the multidisciplinary supervision team, and consensus was reached through iterative discussion and refinement of the thematic map of themes and subthemes.

Adopting an inductive approach means the final themes are strongly linked to the initial data^34^ without requiring the data to fit pre-existing coding frameworks to match the interviewer’s or theoretical analytic preconceptions. Member checking is known to strengthen the reliability of qualitative research findings.^36^ All midwives who participated were invited to take part in member checking and were presented with the final themes and sub-themes generated from analysis. Of the 10 midwives who participated, three midwives responded by email to validate the final themes and sub-themes generated from their interview or focus group transcription.

## Results

Ten midwives participated in the study and Table 1 summarizes their demographic data. Of the 10 participants, three were endorsed midwives working in continuity of care models of midwifery practice. In Australia, endorsed midwives make up 3% of the workforce^37^ and were therefore overrepresented in this sample. Participants were all female with more than 10 years career experience and aged between 35 and 64. One midwife identified as an Aboriginal Australian and over half of the participants were employed in Western Australia.

**Table 1.** Demographic characteristics of participants.

Demographic characteristic	(*n* =)
Age	
35–44	4
45–54	5
55–64	1
Gender	
Female	10
Male	0
Aboriginality	
Yes	1
No	9
Years of experience	
10–15	8
15–20	0
More than 20	2
Location	
Victoria (VIC)	3
New South Wales (NSW)	1
Western Australia (WA)	6
Current employment	
Endorsed midwife	3
*Private Practice - Homebirth (2)*	
*Midwifery Group Practice (1)*	
Midwifery Service Manager	1
Registered Midwife	2
Clinical Midwife	2
Midwife Researcher	1
Midwife Advisor Perinatal Mental Health	1

### Thematic analysis

**Figure 2.**
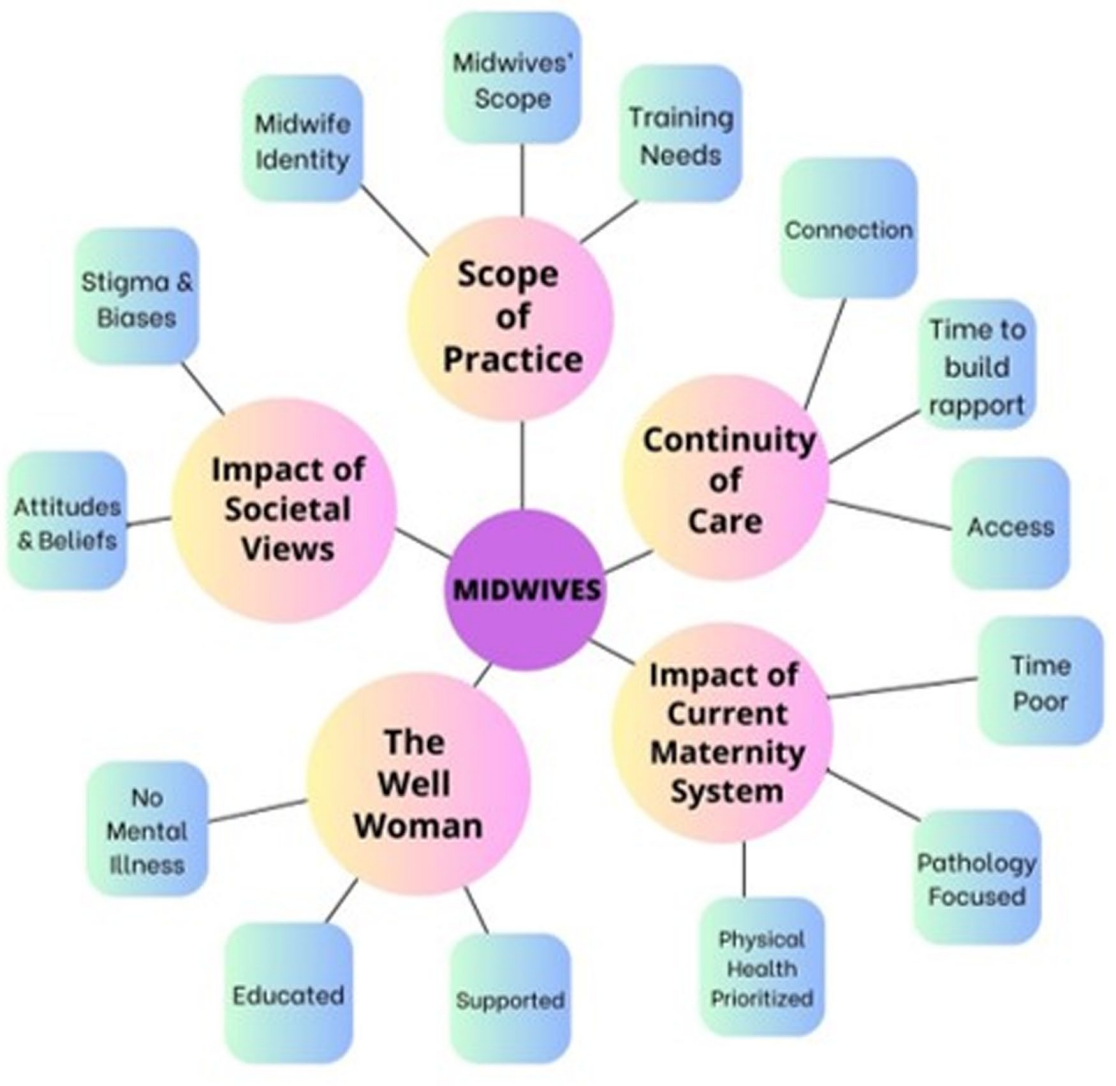
Thematic map of themes and subthemes.

Key themes were generated from the data: The Well Woman; Impact of Societal Views; Scope of Practice; Continuity of Care and Impact of Current Maternity System. A diagram of themes and sub-themes is presented in Figures 1 and 2. Supporting perspectives from interview excerpts are presented in Table 2.

**Table 2.** Summarized perspectives identified by midwives, exemplified by interview excerpts.

Sub-theme	Interview excerpt	Interview participants location in Australia
No mental illness	Emotional wellbeing is connected to country, to identity and when women are dislocated from their country, her spirit will be low. . .women need to feel connected. . . [emotional wellbeing] is individually based, it is not one size fits all.	First Nations midwife, Western Australia
Education	Women with the ability to do her own research, be knowledgeable and empowered to be able to communicate what she needs I think springs to mind as the most important [if she is to have optimal mental health].	Western Australia
Support	I think for women it is about being supported, about being heard and having support from family, friends and maternity care providers. When we feel supported, we have a sense of wellbeing. Women need to have this through pregnancy and birth if they are to be well after the birth journey.	Western Australia
Stigma & bias	The environment around women is a big part of the problem. Socialization of girls teaches us to internalize so much as women. . . it would make a big difference if we could use societal expectations to our advantage instead of our disadvantage.	Western Australia
Attitudes & beliefs	Women’s expectations of perinatal mental health are easy to describe . . . they are thinking about anxiety and depression. Nothing else. . . Women, particularly younger women struggle to normalize the natural ebbs and flows of mood.	Western Australia
	“Perinatal mental health can be a positive thing. We need to spin it and say, how can we increase your perinatal mental health and make you in a better place so that when you have the baby you are not in a lesser space that is not to dismiss anxiety or depression but how about we build up strong mothers so that they can have the buffer. For me strong mothers involve identifying what the women needs even before pregnancy. We just need to recognize it for what it is.”	First Nations midwife, Western Australia
Midwife identity	In terms of midwifery scope around emotional wellbeing, I feel like it is actually a massive part of what being a midwife means . . . we all know midwife means being with woman . . . you are not just physically there with someone, you are there in every aspect. So, the emotional wellbeing of your client is actually really important . . . it is a very intertwined aspect to care.	Western Australia
Training needs	I am not sure you can [teach midwives] to be able to improve the mental health and emotional wellbeing of women. It is either in us or it is not, and I see it every shift because we all have different personalities. The midwives with the type A personality where they want to walk in and immediately need to fix everything, that is what they will do. But what you are talking about [optimizing emotional wellbeing] I think is something that can’t be taught. It is difficult to put my finger on, but I don’t think all midwives have it. No.	Western Australia
Connection	It [emotional wellbeing] is innately in women but because of her life experience, women come to us looking for something different. They know [the public hospital system] is not going to give that to them, so they come to us [private midwives].	New South Wales
Time to build rapport	I think it is important to define that continuity of care and continuity of care provider are different. When we look at the models [optimizing emotional wellbeing] could be achieved by women accessing consistency in terms of how we converse with women and the information we are providing. I think that is achievable in any model. That would be an important thing to target.	Western Australia
Access	The federal government needs to pay for all women to birth where they choose to birth. . . . women with choice will have better outcomes and therefore better mental health.	New South Wales
Time poor	“Antenatal appointments in most hospital-based models of care are set up with timeslots that do not have extra time to be able to have a yarn. There is no quiet space. No softness and comfort. Especially for Aboriginal women, culturally secure spaces are essential if women are to be able to talk [in their own time] about any concerns.”	First Nations midwife, Western Australia
No screening	We do the mental check in during antenatal appointments, I suppose it is not an official thing, but I always ask, how are you going, how are you coping with the pregnancy. But it is not an official thing to ask about no.	Victoria
Pathology focused	“Midwifery care in the metropolitan healthcare system in tertiary and secondary public hospitals is only set up to support someone with mental health issues if they are in crisis mode. . .I don’t think there is a big enough cohort of midwives focused on working to improve mental health and wellbeing. . .a lot of them are very focussed on getting a good outcome for themselves as well as the women and the baby, you know the physical outcome that everyone is alive at the end of labour.”	Western Australia
Physical health prioritized	“Generally, no, because in the time we have in clinic physical health is the primary focus and then if there is any time left over, I might ask about how things are going, do you have any worries?”	Western Australia

### The well woman

Midwives offered definitive descriptions of emotional wellbeing in pregnant women and mothers. Three sub-themes describe perspectives of the Well Woman: No mental illness, Educated, and Supported. Non-Aboriginal midwives working in standard public hospital models of maternity care described recognizing emotional wellbeing in the absence of mental illness. “She [the well woman] has no history [of mental illness] or they have a history but currently they are presenting to you without mental health difficulty.” (Victoria participant) During an online focus group, midwives discussed their experience of working with women and their attitudes toward emotional wellbeing. “Women want to be stress free and don’t want to develop any mental health issues during pregnancy. . .but some people are worried about [developing mental illness in pregnancy].”

Midwives spoke enthusiastically about the role of women accessing education in pregnancy to enhance emotional wellbeing, recognizing this may not be knowledge the women have gained before pregnancy. One perspective was “I think that [optimizing emotional wellbeing] is one aim of antenatal education classes. . . honestly, women should have to do education in pregnancy, not opt in but opt out. Knowledge is such a big part of a [positive] experience.” (Western Australia participant)

The link between emotional wellbeing and women being supported at an interpersonal level outside of the woman-midwife relationship was summarized by a First Nations midwife, identifying the needs of childbearing women. “If I know a woman is feeling low, we need to find out what helps fill them up and make them feel connected. So, I would use my skills to make sure she is feeling connected, loved and cared for, not just tick boxes and do the EPDS [Edinburgh Postnatal Depression Scale] screening.” (Western Australia participant)

### Impact of societal views

Two sub-themes describe the perspectives of barriers to maternal emotional wellbeing for Australian women: Stigma and Bias and Attitudes and Beliefs. “Social media depicts women having babies and looking perfect, gym ready in their sports gear with these newborns but life is not like that.” (Western Australia participant) describing the negative impact societal views have of the experience of postpartum mothers.

There was consensus within the study participants that the orientation of attitudes toward perinatal mental health are central to strengthening emotional wellbeing. Prioritizing conversations may support women and midwives to recognize and build awareness of the difference between mental health and mental illness. One midwife described this was not something she openly discussed with women. “That is an interesting question [asking well women about existing supports to manage mental health], because I’m just wondering now when I think back in the past it is probably not something that I have openly talked about with women.” (Victoria participant)

### Scope of practice

Three sub-themes summarize participant of Scope of Practice: Midwife Identity; Midwives’ Scope and Training Needs. Midwives recognized the importance of emotional wellbeing within the holistic role of midwifery care during focus group discussion stating, “We do have a really integral role, a very significant role and opportunity to empower and optimize in any way we can.” (Victoria participant). A homebirth midwife offered a view that changes in professional identity would support optimal outcomes for women. “A big change [for women to have optimal mental health and emotional wellbeing] would be for midwives to be a separate entity from nurses. We are different. Nurses are medical personnel who work with people who are ill. That is where it needs to start. . .Midwifery needs to be respected as a standalone profession, and women need to understand the difference [between a nurse and a midwife].” (New South Wales participant)

The promotion of mental health and emotional wellbeing was discussed within the focus group confirming “Emotional health and wellbeing I suppose is within our scope” (Western Australia participant) however barriers to working to scope were identified.

“Because of our training, we are very programmed to refer on as soon as we detect something [abnormal]. The multi-disciplinary team can be amazing. . .but I think we can actually do a lot more than we do. Our scope is quite large. . .I feel we haven’t been able to realize our potential in the way midwives are employed at the moment.” (Western Australia participant)

Whilst in the focus group, participants acknowledged their expertise in supporting health states, they described the need for further training specifically around promoting mental health and emotional wellbeing. “Probably what we are lacking is better training in emotional health and wellbeing because we get all the training in anxiety and depression which are the most common pathologies we see. I don’t remember getting any education, support or clinical training around supporting or optimizing emotional health and wellbeing.” (Western Australia participant)

Participants were enthusiastic when discussing optimizing mental health and emotional wellbeing stating, “I think [optimizing mental health and emotional wellbeing] must be done by midwives as we are the biggest part of the journey with women.” (New South Wales participant)

### Continuity of care

Continuity of care was regarded as a potential solution. Three sub-themes describing possible solutions including: Connection; Time and Access.

Endorsed midwives working in private practice offering women opportunities to birth at home described the importance of women connecting and having conversations with her midwife when emotional wellbeing is challenged: “For me it’s quite simple as I can have a conversation because I see my women all the way through until six weeks postpartum. I can refer on if needs be, but they can reach out to me if they are feeling something is not quite right or they need to ask a question.” (Victoria participant)

Building rapport and connection was also discussed by a participant working in midwifery group practice stating “I think [to optimize mental health and emotional wellbeing] there has to be a bit of rapport developed for women to be able to open up fully to you. . .in a continuity model seeing the same person, you develop a relationship over time so that helps.” (Western Australia participant)

The focus group participants discussed and compared experiences of different models of care, recognizing that restricted time is a barrier to optimizing maternal mental health and emotional wellbeing. “Let’s be honest, in an antenatal clinic, I wouldn’t really have time to even ask, in fact if I did a pathology screening like the EPDS and it was abnormal, there is no kind of leeway for it to be abnormal because you are not allocated any time to potentially deal with any of the things that come up which is very, very frustrating” (Western Australia participant)

Participants shared perspectives to address barriers offering definitive solutions toward change confirming “In continuity models where women have continuity of care with a known midwife the overall objective is to strive towards making women’s emotional health and wellbeing a priority. By making it a priority, she has better physical health outcomes as well. . . however models of care like this are not available to all women in Australia.” (Victoria participant)

### Impact of current maternity system

Participants reflected on the role of the maternity system as a barrier to promoting perinatal mental health and wellbeing. Three sub-themes were generated: Time Poor; No Screening and Physical Health Prioritized. Examples from clinical experience of restricted time when providing care to pregnant women were shared. “Definitely not. No is the only answer to that question. . . the demands of the midwifery role in standardised maternity care is not set up to have time [to optimize mental health and emotional wellbeing].” (Western Australia participant)

Screening practices dedicated to identifying mental illness was described. “Apart from the Edinburgh Postnatal Depression Scale, I don’t think there is actually any other thing we really probe for. I am trying to think. . .It is a really great question though because it does make you think, well why don’t we probe a bit more? Why are we not asking those questions?” (New South Wales participant)

Discussions in the focus group shared perspectives that healthcare system change as a possible solution for change. “The way we provide care has a profound effect on our capacity to even recognize emotional wellbeing in women. If we are really, truly dedicated to ensuring the mental and emotional health of women is prioritized, it is almost impossible to continue to provide care in the way we are which is obviously not enough for women. I doubt any midwife no matter how good they are is going to be able to provide women with what they need to improve mental health and emotional wellbeing because everyone is different. We cannot do this in an institutionalized style environment within maternity care.” (Western Australia participant)

Mental health and emotional wellbeing were described as an afterthought to physical health priorities during routine midwifery care. “I worked in a tertiary setting most recently, in antenatal clinic there are all the physical health questions, blood pressure, urine analysis, palp the baby. You do all this in a little booth that isn’t really private at all. How is a woman going to feel safe to be able to share what she is feeling in a space like that? She isn’t.” (First Nations participant, Western Australia)

## Discussion

This study explored Australian midwives’ perceptions of identifying emotional wellbeing within their role as primary maternity care providers. While previous studies have examined midwives’ knowledge, attitudes, and confidence in managing perinatal mental illness, this study shifts attention toward the promotion of mental health and emotional wellbeing as a public health function of midwifery practice. To our knowledge, this is the first Australian study to qualitatively explore and report this in evidence. Therefore, this study also sought to explore midwives’ perspectives of possible solutions to strengthen public health through midwifery practice to support mentally healthy mothers. Our findings highlight several aspects for further discussion.

Midwives are uniquely positioned within healthcare to support mentally healthy mothers^37,38^ as they provide health care across the continuum from pregnancy to the early weeks after birth, thereby securing better outcomes for women and babies.^14^ Midwives in this study described their health promoting role and their willingness and desire to strengthen mental health of women in their care. However, perspectives of what it means for women to be mentally healthy with emotional wellbeing during pregnancy and after birth were offered in the absence of PMADs including anxiety and depression. The sustained use of illness-oriented frameworks within maternity care appears to shape how emotional wellbeing is conceptualized in practice. In this study, midwives frequently defined maternal wellbeing in terms of the absence of perinatal mood and anxiety disorders, reflecting the dominant screening-based approach embedded within current guidelines.^3^ This finding magnifies the call to action to adopt a broader conceptual vision of mental health that recognizes wellbeing as more than the absence of pathology, particularly during the perinatal period. This was attributed in the study to the impact and pressures of the current maternity system, orientation of screening practices and educational preparation focused on detecting risk of mental illness and ongoing employment within a medical model of maternity care. Midwives identified solutions to support and nurture mentally healthy mothers through pregnancy and postpartum maternity care including professional training needs and systemic changes to the way midwives are employed, supporting findings from previous research.^39,40^

Midwives in this study endorsed the benefits available to women accessing continuity of care models where opportunities to optimize mental health and emotional wellbeing exist within relationships of trust between the woman and her midwife.^10^ The benefits of continuity of midwifery care are not new understandings and have been widely studied both in Australia and across the world for over 20 years.^39,40^ Midwives offered perceptions of barriers to identify and support mental health promotion including funding restrictions, limited access to a known midwife and time constraints within standard maternity models where competing priorities between prioritizing mental health and physical health exist. Resource constraints including funding, access and time pressures are reported to have placed significant burden on midwifery care,^41^ recognizing the public health benefits derived from the provision of high-quality maternity care aligned with national strategic policy is not fully realized in Australia.^42^

Endorsement for continuity of midwifery care was described by an Aboriginal midwife centering care around culturally safe, woman-centered conversations to identify individual needs founded in a woman’s connection to her country. Through connection and cultural understanding, identifying emotional wellbeing through yarning with a known midwife.^43^ Aboriginal women are overrepresented in populations experiencing mental illness in the perinatal period; therefore continued investment in continuity models of care for all women birthing in Australia is warranted to prioritize the optimization of maternal mental health and emotional wellbeing.

Midwives described the role of antenatal education as a potential intervention to strengthen maternal mental health for women and their families. Acknowledging mental health education may not be best delivered as a “one size fits all” model, midwives shared no awareness of formal midwifery-led educational opportunities within their workplace. In the absence of national standards to govern the content and provision of antenatal education programs in Australia,^44^ midwives in this study strongly recommended pregnant women access evidence-based education as an enabler of improved mental health. Findings of a recent scoping review highlighted no mental health promotion interventions are offered exclusively to women in Australia.^45^ The review concluded that there is scope to consider mental health promotion interventions in primary care where implementation toward strengthening public health may benefit from engaging the midwifery profession in an educator/facilitator role. The potential feasibility of this is currently unknown in Australia.

Our findings present new evidence to support the potential of the workforce to strengthen public health through supporting mentally healthy mothers. Interpreting these findings through a salutogenic lens highlights an important pattern. Midwives demonstrated strong professional meaningfulness in their desire to promote maternal mental health yet described limited comprehensibility (training and conceptual clarity) and manageability (time, resources, structural support). This imbalance may constrain their capacity to enact a strengths-based mental health promotion role. Strengthening midwives’ knowledge, skills, and structural supports may therefore enhance their own sense of coherence in practice, with potential downstream benefits for women’s emotional wellbeing. The potential impact of midwives with knowledge, skills and resources to strengthen maternal mental health and wellbeing is currently unknown and warrants further research in Australia.

### Strengths and limitations

This study was strengthened by the breadth of participants’ professional backgrounds, years of experience, and inclusion of Aboriginal midwifery perspectives. However, recruitment was limited to midwives practising in Western Australia, Victoria, and New South Wales, which may limit the transferability of findings to other Australian jurisdictions. Participants were recruited using online social media platforms, which represents a further limitation. While social media is increasingly used for recruitment in qualitative research, midwives who do not engage with social media or who are less comfortable with digital technologies may have been excluded, potentially limiting the diversity of perspectives captured. The study was strengthened by a multidisciplinary research team, enabling interpretation of findings through midwifery, mental health, and developmental psychology lenses.

## Significance for public health

This study provides novel evidence regarding Australian midwives’ perspectives of identifying and supporting maternal mental health promotion through midwifery practice. Our findings highlight midwives in Australia identify emotional wellbeing as the absence of mental illness, reflecting the orientation of tertiary midwifery education. Whilst midwives unanimously agreed promoting mental health is within their role and scope of practice, barriers to working with women to optimize mental health were impacted by societal views, priorities, and systemic pressures of time within public hospital models of maternity care and limited access to both publicly funded and private continuity of care models.

Our findings suggest Australian midwives are well-placed but not equipped to promote maternal mental health at present. If we are to realize the potential of our perinatal population, changes to midwifery practice would require midwives to acquire additional knowledge and skills and warrants further research.

## Supplemental Material

sj-docx-1-phj-10.1177_22799036261434100 – Supplemental material for Building healthy beginnings: A qualitative descriptive study of midwives’ role in identifying and promoting mentally healthy mothers in Australia Supplemental material, sj-docx-1-phj-10.1177_22799036261434100 for Building healthy beginnings: A qualitative descriptive study of midwives’ role in identifying and promoting mentally healthy mothers in Australia by Lesley Pascuzzi, Karen Heslop, Helen Skouteris and Zoe Bradfield in Journal of Public Health Research
