# Supplementary material for: Building healthy beginnings: A qualitative descriptive study of midwives’ role in identifying and promoting mentally healthy mothers in Australia
Source: J Public Health Res. 2026 Mar 26;15(1):22799036261434100. doi: 10.1177/22799036261434100 (PMC13031753; doi:10.1177/22799036261434100)
Supplement: sj-docx-1-phj-10.1177_22799036261434100 – Supplemental material for Building healthy beginnings: A qualitative descriptive study of midwives’ role in identifying and promoting mentally healthy mothers in Australia [file sj-docx-1-phj-10.1177_22799036261434100.docx]

Supplementary File 2

Interview Questions.

1. Can you describe to me what you think it means to be emotionally well during the journey through pregnancy to parenthood
   1. Prompt 1 – you mentioned emotional wellness is something you notice in a pregnant women/mother. Can you tell me more about a recent time you saw a woman who you believed was emotionally well?
   2. Prompt 2 – can you describe what you mean by wellbeing when a woman has no signs of anxiety or depression
   3. Prompt 3 – If I was to watch you working with or educating a woman you were working with towards her desired state of mental health and wellbeing, what would I see you doing/saying to her?
2. What solutions do you think are needed to support midwives to work with women towards becoming mentally healthy?
